# Public health and social measures during health emergencies such as the COVID‐19 pandemic: An initial framework to conceptualize and classify measures

**DOI:** 10.1111/irv.13110

**Published:** 2023-03-09

**Authors:** Eva A. Rehfuess, Ani Movsisyan, Lisa M. Pfadenhauer, Jacob Burns, Ramona Ludolph, Susan Michie, Brigitte Strahwald

**Affiliations:** ^1^ Institute for Medical Information Processing, Biometry and Epidemiology LMU Munich Munich Germany; ^2^ Pettenkofer School of Public Health Munich Germany; ^3^ Department of Epidemic and Pandemic Preparedness and Prevention WHO Health Emergencies Programme, World Health Organization Geneva Switzerland; ^4^ UCL Centre for Behaviour Change University College London London UK

**Keywords:** complex interventions, COVID‐19, evidence‐informed decision‐making, non‐pharmacological interventions, pandemic preparedness, systems thinking

## Abstract

**Background:**

Public health and social measures (PHSM) intend to reduce the transmission of infectious diseases and to reduce the burden on health systems, economies and societies. During the COVID‐19 pandemic, PHSM have been selected, combined and implemented in a variable manner and inconsistently categorized in policy trackers. This paper presents an initial conceptual framework depicting how PHSM operate in a complex system, enabling a wide‐reaching description of these measures and their intended and unintended outcomes.

**Methods:**

In a multi‐stage development process, we combined (i) a complexity perspective and systems thinking; (ii) literature on existing COVID‐19 PHSM frameworks, taxonomies and policy trackers; (iii) expert input and (iv) application to school and international travel measures.

**Results:**

The initial framework reflects our current understanding of how PHSM are intended to achieve transmission‐related outcomes in a complex system, offering visualizations, definitions and worked examples. First, PHSM operate through two basic mechanisms, that is, reducing contacts and/or making contacts safer. Second, PHSM are defined not only by the measures themselves but by their stringency and application to specific populations and settings. Third, PHSM are critically influenced by contextual factors. The framework provides a tool for structured thinking and further development, rather than a ready‐to‐use tool for practice.

**Conclusions:**

This conceptual framework seeks to facilitate coordinated, interdisciplinary research on PHSM effectiveness, impact and implementation; enable consistent, coherent PHSM monitoring and evaluation; and contribute to evidence‐informed decision‐making on PHSM implementation, adaptation and de‐implementation. We expect this framework to be modified and refined over time.

## 1 INTRODUCTION

At the beginning of the COVID‐19 pandemic, many of the characteristics of the novel virus SARS‐CoV‐2 were unknown, and neither vaccines nor effective pharmaceutical treatments were available. This placed public health and social measures (PHSM) in the spotlight then and throughout the pandemic.^1^ Although the importance of PHSM is recognized globally, many questions remain.

PHSM—often also referred to as non‐pharmacological interventions in the literature—are intended to reduce the risk and scale of the transmission of infectious diseases, as well as to reduce the burden on health systems, economies and societies. Indeed, PHSM can contribute to pandemic control by reducing the overall number of cases or infection‐related deaths and/or by postponing or lowering infection peaks (‘flattening the curve’). Usually, multiple PHSM are employed in parallel, recognizing that no single measure is sufficient in effectively reducing transmission. PHSM represent ‘complex interventions in complex systems’^2^: As multi‐component measures, they are governed and implemented across multiple levels (international, national, sub‐national) and different sectors (including health, tourism, education, trade, social services). Their effectiveness and impact depend on contextual factors, such as geographical setting, and socio‐cultural aspects, such as societal compliance and trust in authority, as well as the timing of implementation and/or de‐implementation.

During the COVID‐19 pandemic, PHSM have been selected, combined and implemented in different ways across countries. There is considerable variation in how PHSM have been conceptualized and categorized in frameworks, taxonomies or policy trackers,^3^, ^4^ leading to fragmentation in monitoring and evaluation. This heterogeneity in policy and practice as well as research presents a challenge for the effective utilization and evaluation of PHSM and for evidence‐informed guidance on PHSM policy in the current and in future health emergencies.

### 1.1 Objective of this paper

This paper presents an initial conceptual framework of PHSM that (i) offers a shared language and understanding of *how PHSM operate to reduce transmission* (intervention mechanisms); (ii) enables a *comprehensive description* of the measures (what?), their stringency (how?), the population targeted (to whom?), the setting of implementation (where?), related health outcomes (what for?) and broader, usually unintended, consequences; and (iii) takes into account *other factors* that may affect the effectiveness and benefit‐harm balance of measures. This framework addresses PHSM during health emergencies due to an infectious agent, using the COVID‐19 pandemic as the basis for developing the method and as a use case.

This framework is conceptual, not operational. It thus provides a tool for structured thinking, rather than a ready‐to‐use tool for practice. It is, however, intended as the starting point for developing specific tools that meet the needs of distinct user groups and institutions, notably those conducting PHSM research, tracking PHSM and making decisions about PHSM.

This work has been undertaken to support the WHO PHSM initiative to measure the effectiveness and broader impact of PHSM during health emergencies, which seeks to strengthen the global evidence base to inform the development of actionable tools for decision‐makers.^5^ The conceptual model will be further developed through extensive expert consultations.

## 2 METHODS

Our approach to developing the conceptual framework of PHSM was informed by a system‐based logic model template^6^ and a staged approach to logic modelling.^7^ The resulting initial framework will be further advanced.

The multi‐stage development process used (i) a theoretical perspective recognizing PHSM as ‘complex interventions in complex systems’^2^; (ii) literature on existing COVID‐19 PHSM frameworks, taxonomies and policy trackers (subsequently referred to as frameworks); (iii) WHO expertise; and (iv) application to two distinct settings. A graphical overview of this development process can be found in Figure S1.

The development and iterative refinement of the framework categories was facilitated through regular meetings among the entire author team, with results presented through visualizations using an online whiteboard (MIRO).^8^ We held author meetings to work on the ‘big picture’, integrate insights gained across the different inputs and discuss and resolve any conceptual inconsistencies; we also held smaller team meetings to address specific components or aspects.

Informed by a complexity perspective and systems thinking, we used selected additional frameworks to advance specific components, and inform broader decision‐making criteria regarding PHSM, especially the Context and Implementation of Complex Interventions (CICI) framework,^9^ the CONSEQUENT framework for unintended consequences of public health interventions,^10^ the Nuffield Intervention Ladder^11^ and the WHO‐INTEGRATE evidence‐to‐decision framework.^12^

To ensure that the conceptual framework builds on existing literature on COVID PHSM, we searched the websites of selected national and international health organizations, conducted targeted literature searches in Pubmed (using combinations of search terms related to COVID‐19 and PHSM, as well as search terms related to taxonomy, conceptual framework and policy tracker) and consulted with WHO. We mapped the identified frameworks by (i) extracting key elements and (ii) (re)coding these elements into a priori defined classes based on the WHO taxonomy and glossary of PHSM^4^ as well as emerging inductive classes for elements that did not fit the pre‐defined classes.

To identify potential missing elements, ensure fit with the objectives and activities of the WHO PHSM initiative and make the framework understandable and appropriate to users, we engaged with members of the WHO PHSM secretariat and the Methods Working Group of the WHO PHSM Initiative and obtained feedback from the WHO PHSM steering group, including staff members from all six WHO regional offices and across a broad range of departments and disciplinary perspectives.

To ensure its face validity and further advance categories and their components, the author team applied the model to develop classifications of PHSM in two settings, namely, in schools and at points of entry to a country (airports, ports and ground crossings), drawing on existing COVID PHSM frameworks, systematic reviews^13^, ^14^ and guidelines,^15^ as well as lived experience.

## 3 RESULTS

### 3.1 Mapping and coding of existing COVID PHSM frameworks

We found 14 frameworks of COVID PHSM, including some referred to by the developers as taxonomies and policy trackers.^3^, ^4^, ^13^, ^14^, ^16^, ^17^, ^18^, ^19^, ^20^, ^21^, ^22^, ^23^, ^24^, ^25^ Key elements extracted from these frameworks (see Supporting Information) provided a starting point for developing the different categories of our framework and the specific components within these categories. We identified four significant challenges with the identified frameworks. First, the majority lacked a *conceptual foundation* and thus resembled a ‘laundry list of measures’ rather than a coherent and consistent classification system. They also had limited conceptual clarity with individual categories not being mutually exclusive, showed varying degrees of granularity and largely did not clearly consider mechanisms and contexts (i.e., what, how, why and for whom). Second, a *complexity perspective and systems thinking* was lacking, with no attention paid to interactions across different categories or components or to the positive or negative consequences of measures. Third, the identified frameworks were poorly equipped to address the *dynamic nature* of a pandemic and rarely took into account adaptation or de‐implementation of measures. Fourth, most lacked definitions of the measures and other relevant aspects (e.g., stringency), as well as explicit links to decision‐making, rendering many of them *difficult to operationalize* in a consistent manner.

### 3.2 The initial conceptual framework

**FIGURE 1:**
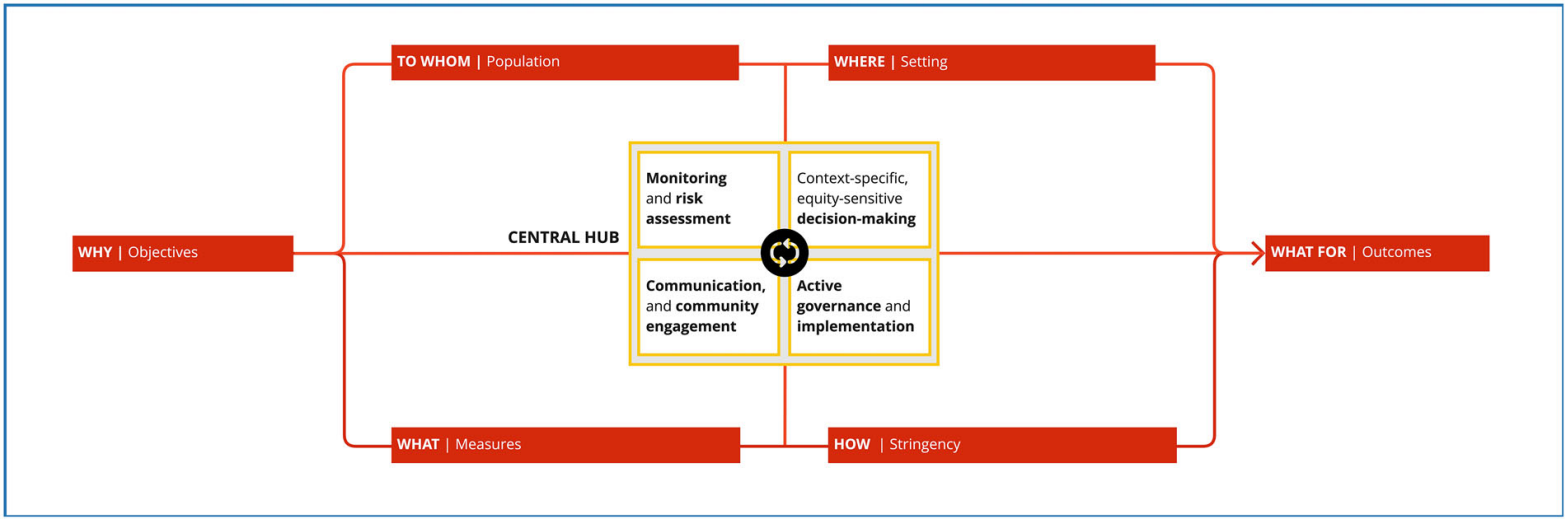
Structure of the initial conceptual framework of public health and social measures during health emergencies.

**FIGURE 2:**
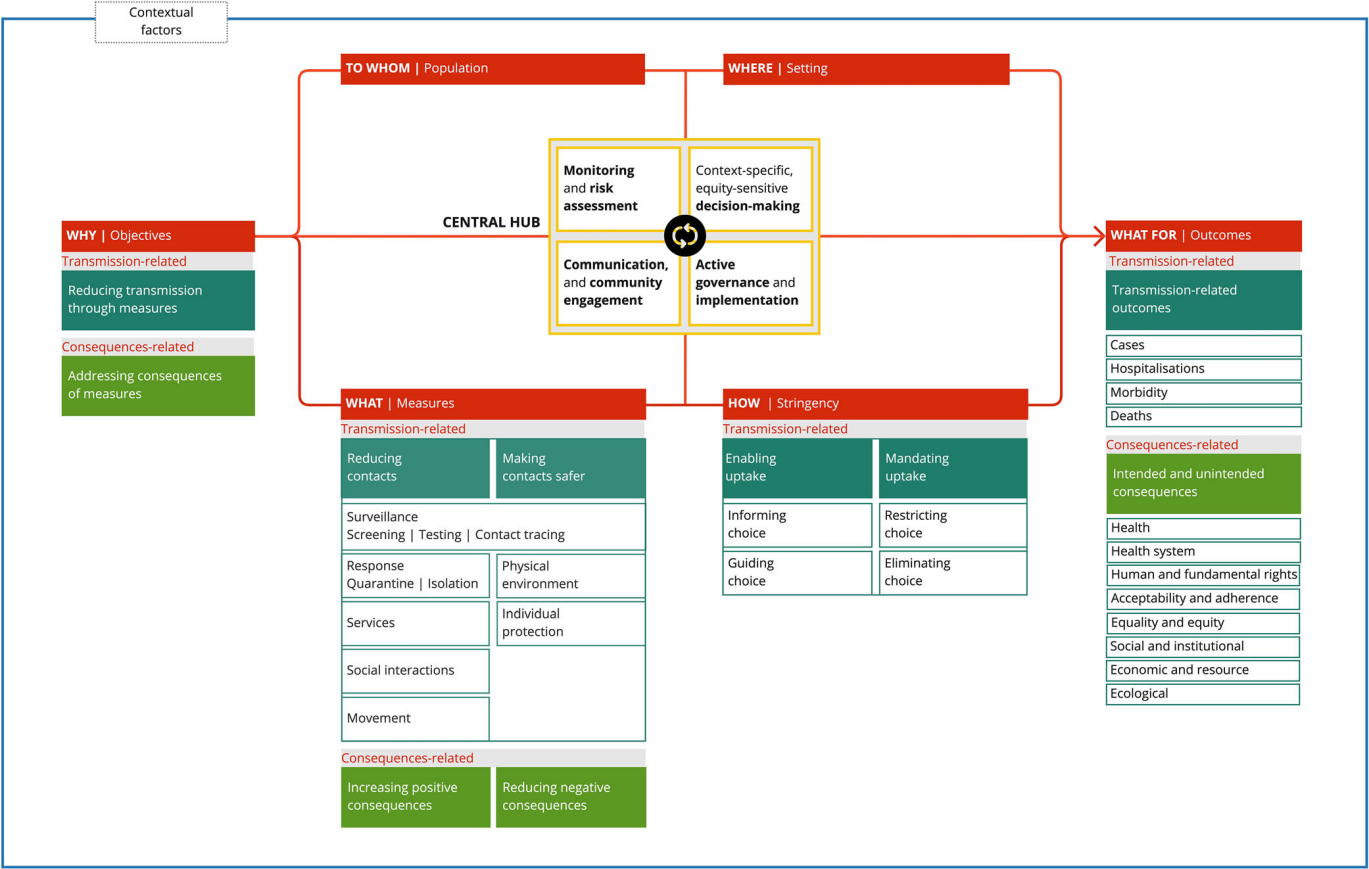
An initial conceptual framework of public health and social measures during health emergencies: framework categories and their components.

Figure 1 presents the structure of the initial conceptual framework. The framework consists of the categories objective, population, setting, measures, stringency and outcomes; a central hub integrates all categories and ensures various critical functions during a health emergency. Each of these categories includes specific components; whereas for some of the categories, the constituent components are well‐elaborated, and the components for the other categories are yet‐to‐be developed through distinct activities of the WHO PHSM initiative. In its structure, the framework thus follows the ‘Russian dolls principle’; that is, it includes a high level of abstraction to maintain simplicity and ensure a ‘big picture view’, but all categories can be further unpacked. Figure 2 presents the initial framework, unpacking the categories objectives, measures, stringency and outcomes. Figure S2 further expands the categories population, setting, contextual factors and context‐specific, equity‐sensitive decision‐making, thus showing all the categories that have been elaborated as part of the work to date.

Importantly, the framework recognizes that (i) all measures have both intended and unintended consequences for health and society and (ii) all categories within the framework are interconnected and interact with each other (even if these interactions are not graphically depicted for better readability). Indeed, any specific PHSM is defined by a combination of the measure itself, the stringency and the population and/or setting targeted.

### 3.3 The underlying reasoning: Presenting intervention mechanisms

**FIGURE 3:**
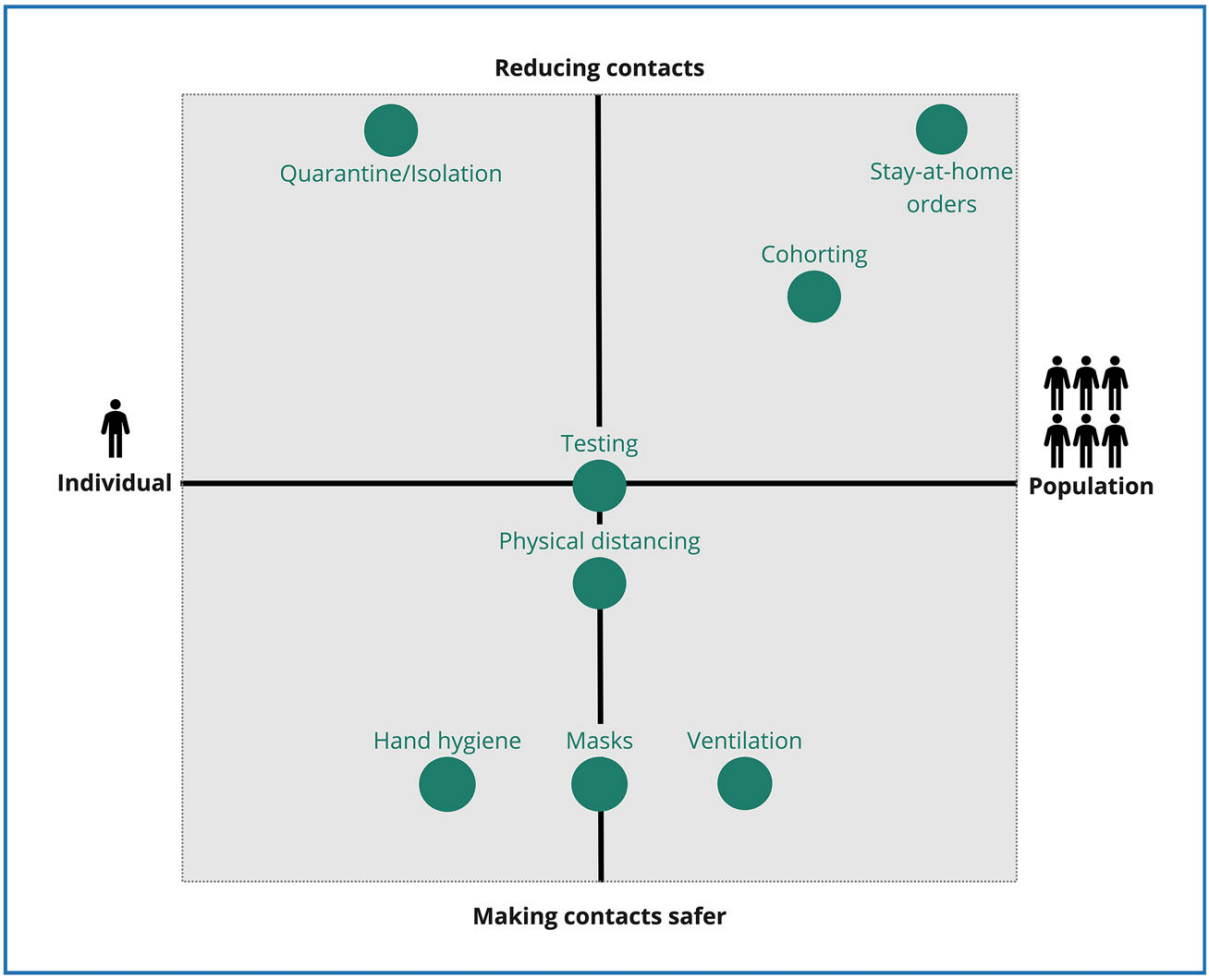
Placement of exemplary public health and social measures (PHSM) according to intervention mechanism and on a spectrum from individual to population agency.

Pathogens are characterized by different modes of transmission, affecting the suitability and effectiveness of distinct PHSM. The current version of the conceptual framework focuses on human‐to‐human transmission, where all PHSM are assumed to operate through two basic mechanisms to reduce transmission: reducing contacts and making contacts safer. For example, quarantining after close contact with an infected person serves to reduce (ideally eliminate) contacts with other individuals; wearing a mask when attending a meeting or going to a supermarket serves to make contacts with others safer. Some PHSM can do both, depending on the perspective adopted. For example, testing can serve to reduce contacts (e.g., restricting hospital access to visitors with an up‐to‐date negative test will reduce the number of visitors); it also serves to make contacts between individuals safer (i.e., contacts in the hospital will only take place between individuals who tested negative). Figure 3 presents widely used categories of PHSM according to their basic mechanism. They fall on a spectrum from measures targeting individuals (e.g., hand hygiene) to population subgroups (e.g., workplace ventilation) to whole populations (e.g., stay‐at‐home orders). Many PHSM can be conceived as individual‐level measures (e.g., mask wearing to self‐protect or to protect others) but show a substantial ‘herd effect’ when widely used at a community and population level (e.g., wide‐spread mask wearing on public transport).

### 3.4 Key categories: Defining PHSM in context

#### 3.4.1 Measures

The framework contains two sets of measures: those that reduce transmission and those that address the consequences of transmission‐related measures (yet to be developed). As described above, for measures reducing transmission, we distinguish between those reducing contacts and those seeking to make contacts safer. The former includes response measures, measures targeting services, social interactions and movement; the latter includes physical environment and individual protection measures. Surveillance measures, depending on the perspective adopted, make use of both intervention mechanisms (see Figure 3). Definitions for each measure are presented in Table 1, accompanied by specific examples.

**TABLE 1:** Measures: Definitions of components and examples.

Mechanism	Measure	Definition	Examples
Measures reducing contacts		*Measures reducing contacts* reduce face‐to‐face/in‐person interactions between individuals or groups of people, thereby reducing the opportunity for transmission‐relevant encounters. They comprise response measures as well as measures to modify services, social interactions and movement of individuals or groups.	
	Response	*Response measures* are implemented in response to an individual case, specific outbreak or rising number of cases in a setting or geographic locality. Classic response measures separate individuals with a confirmed or suspected infection (isolation) or with an increased risk of infection (quarantine) from people at risk of becoming infected. Additional response measures from across all categories of measures (e.g., responsive mask wearing or responsive testing) may be implemented in a time‐limited, short‐duration manner.	Quarantine, isolation, daily testing (responsive only)
	Services	*Measures to modify services* comprise adaptation, cancellation and/or modified timing of services or activities to prevent transmission of an infectious agent.	Closure of schools, closure of non‐essential businesses
	Social interactions	*Measures to modify social interactions* adapt the ways in which individuals and groups of people interact with each other.	Cancellation of large gatherings
	Movement	*Measures to modify movement* adapt the ways in which individuals or groups of people move within and between specific settings and within or across national borders.	Suspension of flights between countries, domestic mobility restrictions
Measures making contacts safer		*Measures making contacts safer* reduce the probability of transmission when people meet in‐person/face‐to‐face. These comprise physical environment measures as well as individual protection measures.	
	Physical environment	*Physical environment measures* operate by directly reducing the exposure of individuals to an infectious agent and/or by enabling healthy behaviours of individuals. They adapt the physical infrastructure (‘hardware’) through modifications to or re‐purposing of the infrastructure and also comprise the appropriate maintenance of existing or newly set up infrastructure.	Physical barriers, ventilation, air purifier, soap and disinfectant provision
	Individual protection	*Individual protection measures* comprise personal protective equipment as well as specific behaviours that reduce the risk of individuals transmitting and/or contracting an infectious agent.	Masks, face shields, gloves, hand‐washing
Both	Surveillance	*Surveillance measures* test or screen individuals and/or groups of people. These make contacts safer by identifying potentially infected and/or infectious individuals and, at the same time, reduce contacts between infected individuals and individuals at risk of being infected. They comprise strategies to test symptomatic individuals or contact persons, to screen an asymptomatic group of people (routinely or in a time‐limited, short‐duration manner in response to an outbreak) or to routinely test a fraction of a certain population to identify potential outbreaks.	Surveillance testing, diagnostic testing, routine screening

#### 3.4.2 Stringency

This category describes the level of strictness with which measures are implemented; it thus primarily refers to the vigour of government action and relates to the extent of individual agency and autonomy. It distinguishes between enabling uptake—empowering and supporting people with regard to certain activities/behaviours by informing choice or guiding choice—and mandating uptake—officially requiring people to take up certain activities/behaviours by restricting choice or eliminating choice. It retains the notion of degree of intrusiveness described in the Nuffield intervention ladder^11^ but compresses its multiple rungs into fewer levels. Importantly, stringency relates to the nature of measures rather than to the means by which they are enacted (e.g., fines to make people obey a mandate). Table 2 provides definitions and examples for each of four levels of stringency.

**TABLE 2:** Stringency: Definitions of components and examples relating to surveillance measures in the school setting.

	Stringency	Definition	Examples
Enabling uptake		*Enabling uptake* serves to empower and support people with regards to certain activities/behaviours.	
	Informing choice	*Inform* choice regarding certain activities/behaviours and/or recommend a given activity/behaviour	Providing information to students and school staff on where to get tested
	Guiding choice	*Guide* choice regarding certain activities/behaviours through enabling measures and/or financial and non‐financial incentives or disincentives	Providing tests to students and school staff free of charge
Mandating uptake		*Mandating uptake* officially requires people to take up certain activities/behaviours.	
	Restricting choice	*Restrict* choice regarding certain activities/behaviours thereby strongly promoting these activities/behaviours but offering limited alternative activities/behaviours	Allowing access to school premises to tested or vaccinated school staff
	Eliminating choice	*Eliminate* choice regarding certain activities/behaviours thereby determining people's activities/behaviours and offering no alternative activities/behaviours (i.e., *any alternative activities/behaviours are associated with extremely high costs*)	Allowing access to school premises to school staff with proof of a negative test only

#### 3.4.3 Outcomes

These comprise those directly related to transmission (i.e., cases, hospitalizations, morbidity during and/or post‐infection and deaths) as well as less direct and broader unintended or intended consequences for health and society, informed by the CONSEQUENT framework.^10^ These consequences may be positive (co‐benefits, e.g., reduced air pollution due to mobility restrictions) or negative (adverse effects, e.g., increased socio‐economic inequality in educational outcomes due to school closures); these could also be described as spillover effects of an intervention.

#### 3.4.4 Setting

This category refers to the specific physical location, in which the intervention is put into practice and interacts with context and implementation.^9^ It comprises 13 specific settings that were drawn up from settings identified in existing COVID PHSM frameworks and subsequently clustered, summarized and complemented by the literature or experts.

#### 3.4.5 Population

This category defines populations according to (i) their susceptibility to infection (e.g., immune status due to prior infection or vaccination) and/or their susceptibility to severe health consequences (e.g., due to age or pre‐existing conditions), (ii) their exposure to infection (e.g. due to living or working conditions) and/or (iii) their susceptibility to and/or experience of negative consequences (e.g., as a result of lower socioeconomic status).

#### 3.4.6 Context

All categories and components of the framework depend on contextual factors. Context reflects a set of characteristics and circumstances that interact with and facilitate or constrain the measures and their implementation; context can be structured according to the seven domains of the CICI framework (e.g., socio‐cultural context and political context), supplemented with the additional domain ‘informational context’, and operates in space and over time.^9^

Numerous *additional factors* influence the choice, design and implementation of measures and thus whether and to what extent different outcomes occur.

### 3.5 The central hub: Connecting all elements

The central hub of the framework connects and integrates all elements of the framework and ensures four critical functions during a health emergency, that is, monitoring and risk assessment, communication and community engagement, context‐specific, equity‐sensitive decision‐making and active governance and implementation. It can facilitate interlinkages with other efforts, for example, with regard to implementation of PHSM or decision‐making about PHSM or with medical countermeasures such as the development and introduction of vaccines and pharmaceutical interventions.

*Monitoring and risk assessment* encompasses monitoring the development of the health emergency, tracking PHSM and conducting integrated risk assessments of the situation. *Communication and community engagement* seeks to set up an information ecosystem to build and maintain trust, offer reliable information and manage the infodemic. *Context‐specific, equity‐sensitive decision‐making* interlinks with existing decision‐making frameworks that bring together evidence and values in a structured manner.^12^ Equity‐sensitive reflects the fact that there are important inequalities—and often inequities—in the risk of becoming infected and/or of experiencing severe health or broader consequences and/or in the ability to adopt a recommended or mandated PHSM.^26^ Context may refer to the overall context (e.g., geography, season and phase of pandemic) or to a specific decision‐making context (e.g., decisions about PHSM in a local institution, for a whole country or within an international organization). *Active governance and implementation* refers to the coordination and responsibilities across different sectors and levels of government and institutions, notably including new laws, regulations or processes that need to be put into place to facilitate the effective implementation and, where applicable, enforcement of PHSM.

### 3.6 Applying the initial conceptual framework to measures for schools and international travel

We applied the framework to two specific settings: schools and points of entry to a country (airports, ports and ground crossings). To develop the classifications, we used measures and levels of stringency that were practised and/or researched during the COVID‐19 pandemic, supplemented with those that are conceivable but may not have been put into practice.

For schools, we defined the aim of measures to maintain schools open while minimizing the risk of infection in the school setting. Stringency refers to the maintenance of essential school services as well as other school activities in‐person for a majority of students. The focus of these measures is on students, but other populations—headteachers, teachers, support staff and parents—are also considered. Essential school services encompass education across the full range of subjects, school health services and school meals, as well as travel to and from school. Table S1 depicts our proposed classification of measures for schools during COVID‐19.

For international travel, the aim of the measures is to reduce the risk of transmission through or during travel between countries via air, land or sea, thereby avoiding or delaying importations/exportations of cases. Stringency refers to the ability of individuals or groups to freely travel between countries, as well as to freely use a range of services (e.g., meals) and opportunities (e.g., having accompanying relatives at the departure/arrival areas) during travel and at the point of entry. Table S2 depicts our proposed classification of measures for international travel and points of entry.

These applications showed that the conceptualization worked well. It surfaced some conceptual challenges that were subsequently resolved. The applications also provided input towards refining definitions for distinct measures and levels of stringency.

## 4 DISCUSSION

### 4.1 Key findings

This paper presents an initial conceptual framework of PHSM during health emergencies due to an infectious agent. This offers a methodologically underpinned conceptual basis of PHSM: First, all PHSM operate through two basic mechanisms to reduce human‐to‐human transmission, that is, reducing contacts and/or making contacts safer. Second, specific PHSM are not only defined by the measures themselves—they depend on their interplay with stringency and the populations and settings targeted. Third, PHSM—the choice of distinct measures and levels of stringency, as well as the resulting benefit‐harm‐balance—are shown to be influenced by a broad range of contextual factors. This framework is a basis for others to use and, in doing so, to suggest modifications and refinements.

### 4.2 Strengths and limitations

To our knowledge, this conceptual framework represents the only available PHSM framework that has used an explicit and robust development process. We used selected published frameworks (literature input) as a starting point and coded their contents but did not conduct systematic literature searches aiming to identify all existing PHSM frameworks. A multipronged, iterative approach to framework development was deemed more suitable given the ongoing pandemic and the need to act compared with an approach that could take years in making with a very comprehensive scoping phase. We advanced the selected frameworks through applying a complexity perspective and systems thinking (theory input). We recognize that other literature and theoretical approaches may help to refine and elaborate this framework. Expert input came through collaboration with WHO; engagement of a broader range of experts from policy and academia as well as methodologists across different disciplines in the context of the WHO PHSM initiative will further advance the framework. The two applications used as a face validity check and suggesting good applicability during the COVID‐19 pandemic represent a start but further face validity checks in additional settings will add value. The ‘stress test’ for the framework will be its ‘real‐life’ application, for example, in an empirical study on the effectiveness, impact and implementation of any given PHSM.

Although the framework is designed to apply to all types of infectious agents and modes of human‐to‐human transmission, we have only used SARS‐CoV‐2/COVID‐19‐related literature in the development process and have only applied it during the COVID‐19 pandemic. The characteristics and mode of transmission of any given pathogen will impact the suitability and effectiveness of different PHSM. The further development of the framework seeks to realize a multiple hazard approach, potentially extending the framework to the context of vector‐ and water‐borne diseases, if feasible and useful. Widening the evaluation of the framework's range of applicability will provide information about its generalizability across populations, settings and type of health threat.

Vaccination is the process or act of immunizing individuals against a specific infectious agent, offering protection against severe consequences of infection and/or against infection. Measures to increase vaccination, while they can be considered PHSM, were excluded from this framework because vaccines are often unavailable during a health emergency and/or are developed, delivered and decided upon through separate mechanisms. The framework thus complements efforts to encourage appropriate uptake of vaccines and pharmaceutical interventions, whether taken prophylactically or as treatment. Where researchers or decision‐makers wish to treat PHSM and vaccination in a more integrated manner, the framework can be modified to accommodate this.

### 4.3 What this conceptual framework can do and cannot do

This initial conceptual framework reflects our current understanding of how PHSM are intended to achieve transmission‐related outcomes in context, offering visualizations, definitions and worked examples. With reference to Nilsen 2020,^27^ we consider this to be a determinant framework, that is, a framework that seeks to understand and/or explain under which circumstances which measures lead to which intended or unintended outcomes. It can be used in a *flexible* manner; that is, one might use the population element as an entry point, focus on a given setting or use a specific measure as a starting point. While its current graphical presentation is static, the framework is *sensitive to changes* in all of its elements, be it developments in the pandemic itself, a new measure becoming available or changes in the decision‐making context.

This framework *lacks detail with regard to conducting empirical research or modelling on PHSM*. It does, however, provide the elements to be considered for research design, data collection and analysis, as well as interpretation of findings. The framework is also *not an implementation framework*. Although it outlines how combinations of measures and levels of stringency may lead to transmission‐related outcomes and broader consequences, the contextual factors influencing implementation are not fully elaborated. The framework by itself also *does not represent a PHSM tracking system*. Our applications to measures for schools (Table S1) and international travel (Table S2), however, illustrate how a comprehensive monitoring system could be developed. While its central hub signals decision‐making as a critical function, this framework is also *not a decision‐making framework*, yet it can systematically guide the development of decision support tools, thereby helping to increase precision in PHSM policies and their implementation.

In summary, the framework represents the starting point for all further activities within the WHO PHSM initiative. In our view, it also provides a foundation for many other research, policy and practice activities to develop and implement more effective PHSM, during the COVID‐19 pandemic and future health emergencies, as recommended by the Lancet Commission on lessons from the COVID‐19 pandemic.^1^ It is intended to (i) facilitate coordinated, multi‐method and interdisciplinary research on PHSM effectiveness, impact and implementation; (ii) enable a comprehensive and nuanced description and documentation of PHSM towards monitoring and evaluation; and (iii) contribute to evidence‐informed decision‐making on PHSM implementation, adaptation and de‐implementation. We look forward to receiving feedback on how to advance this conceptual framework.

## AUTHOR CONTRIBUTIONS

**Eva A. Rehfuess:** Conceptualization; data curation; formal analysis; methodology; validation; visualization; writing – original draft; writing – review and editing. **Ani Movsisyan:** Conceptualization; data curation; formal analysis; methodology; validation; visualization; writing – original draft; writing – review and editing. **Lisa M. Pfadenhauer:** Conceptualization; data curation; formal analysis; methodology; validation; visualization; writing – original draft; writing – review and editing. **Jacob Burns:** Conceptualization; data curation; formal analysis; methodology; validation; visualization; writing – original draft; writing – review and editing. **Ramona Ludolph:** Conceptualization; data curation; formal analysis; methodology; visualization; writing – original draft; writing – review and editing. **Susan Michie:** Conceptualization; data curation; formal analysis; visualization; writing – original draft; writing – review and editing. **Brigitte Strahwald:** Conceptualization; data curation; formal analysis; methodology; validation; visualization; writing – original draft; writing – review and editing.

## CONFLICT OF INTEREST STATEMENT

AM, LMP, JB, EAR and BS report having received institutional funding from the German Ministry for Education and Research towards the COVID‐19 Evidence Ecosystem (CEOsys) project, which facilitated systematic review work on school measures and international travel measures. EAR additionally reports being a member of the Scientific Advisory Board of the Robert Koch Institute, which offered guidance on PHSM during the pandemic in Germany, and of the WHO Regional Office for Europe's Technical Advisory Group on Schooling during COVID‐19; she also reports being a member of the methods working group of the WHO PHSM Initiative. RL reports being involved with setting up and implementing the WHO PHSM Initiative. SM reports being a member of the methods working group of the WHO PHSM Initiative, having participated in the UK Government's Scientific Advisory Group in Emergencies (SAGE) and in Independent SAGE, and being a member of the Lancet Covid‐19 Commission's Public Health Taskforce.

## DISCLAIMER

The authors alone are responsible for the views expressed in this article, and they do not necessarily represent the views, decisions or policies of the institutions with which they are affiliated.

### PEER REVIEW

The peer review history for this article is available at https://publons.com/publon/10.1111/irv.13110.

## Supporting information


Included COVID PHSM taxonomies, frameworks and policy trackers with referencesMapping of contents of included COVID PHSM taxonomies, frameworks and policy trackers
**Figure S1.** Development process towards initial conceptual framework of PHSM.
**Figure S2.** An initial conceptual framework of public health and social measures during health emergencies: framework categories and their components (population, setting, contextual factors and context‐specific, equity‐sensitive decision‐making expanded).
**Table S1.** Classification of measures for schools during COVID‐19.
**Table S2.** Classification of measures for international travel and points of entry during COVID‐19

## Data Availability

The data contributing to the development of the conceptual framework comprise existing PHSM frameworks, including taxonomies and policy trackers, identified from websites of organizations, targeted literature searches in Pubmed and consultations with WHO. The references for all of these PHSM frameworks, taxonomies and policy trackers as well as their main contents are available in the supplementary material. The coding of these data against the conceptual framework of PHSM will be made available to others upon reasonable request. Additionally, data generated by our application of the conceptual framework to two settings, schools and international travel, led to further refinement of the framework. These applications are provided in full in the supplementary material.
