# Supplementary material for: Public health and social measures during health emergencies such as the COVID‐19 pandemic: An initial framework to conceptualize and classify measures
Source: Influenza Other Respir Viruses. 2023 Mar 9;17(3):e13110. doi: 10.1111/irv.13110 (PMC9996427; doi:10.1111/irv.13110)
Supplement: Supplementary file 1 — Included COVID PHSM taxonomies, frameworks and policy trackers with referencesMapping of contents of included COVID PHSM taxonomies, frameworks and policy trackers Figure S1. Development process towards initial conceptual framework of PHSM. Figure S2. An initial conceptual framework of public health and social measures during health emergencies: framework categories and their components (population, setting, contextual factors and context‐specific, equity‐sensitive decision‐making expanded). Table S1. Classification of measures for schools during COVID‐19. Table S2. Classification of measures for international travel and points of entry during COVID‐19 [file IRV-17-e13110-s001.docx]

**Public health and social measures during health emergencies such as the COVID-19 pandemic: an initial framework to conceptualize and classify measures**

Eva A. Rehfuess Ph.D., Ani Movsisyan D.phil., Lisa M. Pfadenhauer Ph.D., Jacob Burns M.Sc., Susan Michie D.phil., Ramona Ludolph, Ph.D., Susan Michie, D.phil., Brigitte Strahwald M.Sc.

## **Supplementary material**

**Table of Contents**

- Included COVID PHSM taxonomies, frameworks and policy trackers with references
- Mapping of contents of included COVID PHSM taxonomies, frameworks and policy trackers
- **Figure S1** Development process towards initial conceptual framework of PHSM
- **Figure S2** An initial conceptual framework of public health and social measures during health emergencies: framework categories and their components (population, setting, contextual factors and context-specific, equity-sensitive decision-making expanded)
- **Table S1** Classification of measures for schools during COVID-19
- **Table S2** Classification of measures for international travel and points of entry during COVID-19

**Included COVID PHSM frameworks, including taxonomies and policy trackers, with references**

- World Health Organization (WHO) taxonomy of public health and social measures (PHSM) ^1^
- COVID-END taxonomy of COVID-19 public-health measures, clinical management, health-system arrangements, and economic and social responses ^2^
- Robert Koch Institute (RKI) Control COVID ^3^
- Complexity Science Hub COVID-19 Control Strategies List (CCCSL) ^4^
- Oxford COVID-19 Government Response Tracker ^5^
- CoronaNet COVID-19 Government Response Event Dataset ^6^
- Health Intervention Tracking for COVID-19 (HIT-COVID) ^7^
- European Center for Disease Control (ECDC) Taxonomy ^8^
- ACAPS COVID-19 Government Measures Dataset ^9^
- COVID-19 Hierarchy of Control ^10^
- Polisena et al (2021) ^11^
- Wang and Mao (2021) ^12^
- Travel measures review (Burns et al. 2021) ^13^
- School measures review (Krishnaratne et al. 2021) ^14^

**References**

1. World Health Organization (WHO). Measuring the effectiveness and impact of public health and social measures. 2022. https://www.who.int/activities/measuring-the-effectiveness-and-impact-of-public-health-and-social-measures (accessed 27/07 2022).

2. Lavis JN. COVID-END taxonomy of public-health measures, clinical management of COVID-19, health-system arrangements, and economic and social responses. 2021 Last updated: 14 April 2022; Hamilton, Canada: McMaster Health Forum; 2021.

3. Robert Koch Institute (RKI). ControlCOVID: Optionen zur stufenweisen Rücknahme der COVID-19-bedingten Maßnahmen bis Ende des Sommers 2021. In: Robert Koch Institute (RKI), editor. Berlin, Germany: Robert Koch Institute (RKI),; 2021.

4. Desvars-Larrive A, Ahne V, Álvarez S, et al. CSH COVID-19 Control Strategies List (CCCSL): Glossary of Codes. 2020. https://github.com/amel-github/covid19-interventionmeasures/blob/master/CCCSL_Glossary%20of%20codes.docx (accessed 21/09/2022.

5. Hale T, Angrist N, Goldszmidt R, et al. A global panel database of pandemic policies (Oxford COVID-19 Government Response Tracker). *Nature Human Behaviour* 2021; **5**(4): 529-38.

6. Cheng C, Barceló J, Hartnett AS, Kubinec R, Messerschmidt L. COVID-19 Government Response Event Dataset (CoronaNet v.1.0). *Nature Human Behaviour* 2020; **4**(7): 756-68.

7. Zheng Q, Jones FK, Leavitt SV, et al. HIT-COVID, a global database tracking public health interventions to COVID-19. *Scientific Data* 2020; **7**(1): 286.

8. European Centre for Disease Prevention and Control (ECDC). Guidelines for the implementation of non-pharmaceutical interventions against COVID-19. European Centre for Disease Prevention and Control Solna, Sweden; 2020.

9. ACAPS Office. COVID-19 Government Measures Dataset. https://www.acaps.org/covid-19-government-measures-dataset27/07/2022).

10. Cornell University. COVID-19 Hierarchy of Controls. 2022. https://ehs.cornell.edu/campus-health-safety/occupational-health/covid-19/covid-19-hierarchy-controls (accessed 27/07/2022.

11. Polisena J, Ospina M, Sanni O, et al. Public health measures to reduce the risk of SARS-CoV-2 transmission in Canada during the early days of the COVID-19 pandemic: a scoping review. *BMJ Open* 2021; **11**(3): e046177.

12. Wang D, Mao Z. A comparative study of public health and social measures of COVID-19 advocated in different countries. *Health Policy* 2021; **125**(8): 957-71.

13. Burns J, Movsisyan A, Stratil JM, et al. International travel‐related control measures to contain the COVID‐19 pandemic: a rapid review. *Cochrane Database of Systematic Reviews* 2021; (3).

14. Krishnaratne S, Littlecott H, Sell K, et al. Measures implemented in the school setting to contain the COVID‐19 pandemic: a rapid review. *Cochrane Database of Systematic Reviews* 2022; (1).

**Mapping of contents of included COVID PHSM frameworks, taxonomies and policy trackers**

World Health Organization (WHO) taxonomy of public health and social measures (PHSM)

| **Category** | **Sub-category** |
| --- | --- |
| **Environmental measures** | ***- Cleaning and disinfecting surfaces and objects - Improving air ventilation - Increasing room humidification*** |
| **Biological measures** | ***- Using antibodies for prevention - Using vaccines for prevention*** |
| **Drug-based measures** | ***- Using medications for prevention*** |
| **Individual measures** | ***- Performing hand hygiene - Limiting face touching - Performing respiratory etiquette - Wearing a mask - Using other personal protective equipment - Physical distancing*** |
| **Social and physical distancing measures** | ***- School measures***   *-- Adapting  -- Closing* ***- Offices, businesses, institutions and operations***   *-- Adapting  -- Closing* ***- Gatherings, businesses and services***   *-- Restricting private gatherings at home  -- Cancelling, restricting or adapting private gatherings outside the home  -- Cancelling, closing, restricting or adapting public gatherings outside the home*  *-- Cancelling, restricting or adapting mass gatherings* ***- Special populations***  *-- Shielding vulnerable groups  -- Protecting populations in closed settings  -- Protecting displaced populations* ***- Domestic travel***   *-- Suspending or restricting movement  -- Stay-at-home order  -- Restricting entry  -- Closing internal land borders* |
| **International travel measures** | ***- Providing travel advice or warning - Restricting visas - Restricting entry - Restricting exit - Entry screening and isolation or quarantine - Exit screening and isolation or quarantine - Suspending or restricting international flights - Suspending or restricting international ferries or ships - Closing international land borders*** |
| **Surveillance and response measures** | ***- Detecting and isolating cases***  -- Passive case detection  -- Active case detection  -- Isolation ***- Tracing and quarantining contacts***  -- Contact tracing  -- Quarantine |

COVID-END taxonomy of COVID-19 public-health measures, clinical management, health-system arrangements, and economic and social responses

| **Category** | **Sub-category** |
| --- | --- |
| **Clinical management of COVID-19 and pandemic-related health issues** | ***- Clinical treatment of C*OVID-19**  -- Assessing most important prognostic factors  -- Screening and testing for COVID-19 (see public-health measures)  -- Drugs to treat COVID-19  -- Anti-virals (see list of drugs)  --- Other antimicrobials (antibiotics, antimalarials, antiparasitics)  --- Anti-inflammatories  --- Kinase inhibitors  --- Corticosteroids  --- Biologics  --- Other  -- Blood products  --- Convalescent plasma  --- Hyperimmune immunoglobin  -- Ventilation for COVID-19  --- Invasive ventilation  --- Non-invasive ventilation  -- Proning  -- Other treatments for COVID-19  -- Other aspects of critical care for COVID19  --- Management of cardiovascular complications  --- Management of renal complications  --- Management of respiratory complications  --- Management of other complications  -- Community-based treatment of COVID-19 and community-based home monitoring  -- Complementary and alternative therapies  ***- Management of COVID-19 with a syndemic orientation***  ***- Treatment of post-COVID conditions***  -- Treatment of long COVID-19 symptoms  -- Treatment of multi-organ effects  -- Treatment of the effects of COVID-19 treatment or hospitalization  -- Treatment of COVID-19 sequelae  ***- Health promotion more generally***  -- Eating healthy food  -- Avoiding or minimizing unhealthy behaviours like smoking or excessive alcohol intake  -- Remaining physically active  -- Staying socially connected  ***- Prophylaxis for COVID-19***  -- Drugs to prevent severe COVID-19 infection  ***- Clinical management of pandemic-related impacts***  ***- Interrupted management of other types of urgent care***  ***- Interrupted management or poor self-management of chronic conditions***  ***- Management of COVID-19 alongside other infectious diseases***  ***- Management considerations for chronic and other existing health conditions***  -- Chronic conditions  -- Cancer  -- Other conditions  ***-*** ***Burn-out and trauma in essential workers***  -- Psychological support  -- Burn-out care  -- Trauma-informed care  ***- Mental health and addiction issues related to the pandemic response***  -- Remote management of existing conditions  -- Management of pandemic-related mental health conditions  ***- Reproductive care for patients with COVID-19***  -- Antenatal care  -- Childbirth  -- Post-partum care  -- New-born care  -- Contraception  -- Termination services  ***- Domestic and gender-based violence related to the pandemic response (see economic and social responses)*** |
| **Economic and social responses** | ***- Transportation***  -- Quarantining travellers  -- Public transportation rules  -- Private transportation restrictions  -- Tourism planning for ‘return to normal’  ***- Housing***  -- Homeless shelters  -- Other congregate living environments  -- Housing alternatives when quarantine or physical distancing is needed  ***- Infrastructure***  -- Broadband internet access Cyber-security protocols for governments and businesses (see financial protection  for protecting citizens from financial scams)  -- Green-space re-allocations to accommodate physical distancing  -- Road-space re-allocations to accommodate physical distancing  -- Domestic production capacity for critical supplies  ***- Natural resources***  -- Price collapses  -- Distribution difficulties  ***- Public safety and justice***  -- Curfews  -- Enforcement of public-health measures  -- Public demonstrations  -- Police work in pandemics  -- Prisons  ***- Recreation***  -- Public spaces like parks  -- Private spaces like gyms  ***- Children and youth services***  ***- Citizenship***  -- Community engagement  -- Civil-rights violations  -- Elections  ***- Climate action***  -- Climate-action focused economic stimulus  ***- Community and social services***  -- Shopping and other services for socially isolated individuals  -- Religious services restrictions (e.g., church, mosque or synagogue)  -- Supports for community resilience  ***- Culture and gender***  -- Stigma reduction  -- Domestic and gender-based violence reduction  -- Arts and cultural institutions  -- Religious institutions and practices  ***- Economic development and growth***  -- Economic resilience  -- Targeted support to most affected industries  -- Interest rate reductions  -- Interest-free or -reduced loans to businesses  -- Revolving credit lines  -- Corporate bond buying (by government)  -- Government bond buying (by central banks)  -- Rent relief for businesses (by government)  -- Debt relief for businesses (by government)  -- Debt relief for governments (e.g., by IMF)  -- Tax deferral for businesses  ***- Education***  -- Online instruction  -- Student supports  -- Instructor supports  -- Classroom changes  -- School changes  -- Skill re-development programs  -- Service planning for ‘return to normal’  ***- Employment***  -- Worker supports  -- Workplace changes  -- Building changes  -- Service planning for ‘return to normal’  ***- Energy and supply***  ***- Environmental conservation***  -- Fire bans due to limitations in and risk for fire-fighting personnel  ***- Financial protection***  -- Income replacement  -- Wage subsidies for essential workers  -- Rent deferral for citizens  -- Debt relief for citizens  -- Tax deferral for citizens  -- Financial-scam prevention  -- Broader consumer protection  ***- Food safety and security***  -- Agricultural processes  -- Food processing plant design  -- Food transportation adjustments  -- Food shopping changes  -- Household food security  -- Food handling practices  ***- Government services***  -- Transitioning to e-services |
| **Health-system arrangements: Financial arrangements** | ***- Financing health services***  ***- Funding organizations***  ***- Remunerating providers***  -- New or adjusted fee codes for virtual care  -- Income replacement when virtual care is not possible (at the same scale)  ***- Purchasing products and services*** |
| **Health-system arrangements: Governance arrangements (who can make what decisions)** | ***- Consumer and stakeholder involvement***  ***- Professional authority***  -- Licensure changes to accommodate out-of-jurisdiction or retired health workers  ***- Commercial authority***  *--* Technology approvals, public-private partnerships  ***- Organizational authority***  -- Limits of number of staff sent in  -- Ownership |
| **Health-system arrangements: Cross-cutting system arrangements** | ***- Approach to COVID-19 vaccine roll-out***  -- Securing and distributing a reliable supply of vaccines and ancillary supplies  -- Allocating vaccines and ancillary supplies equitably  -- Communicating vaccine-allocation plans and the safety and effectiveness of vaccines  -- Administering vaccines in ways that optimize timely uptake  -- Surveillance, monitoring, evaluation and reporting  ***- Approach to population-health management for COVID-19 and for those whose care is disrupted by COVID-19***  -- Segmenting the population into groups with shared health and social needs  -- Re-designing care pathways and in reach and out-reach services  -- Addressing barriers to implementation of pathways and services  -- Addressing cultural safety in the implementation of pathways and services  -- Maintaining gains made in population health management (e.g., population segmentation, virtual care) and  spreading and scaling them |
| **Health-system arrangements: Delivery arrangements** | ***- Overall service planning for COVID-19 preventing and treatment***  *--* Leveraging existing health-system arrangements  ***- Service planning for COVID-19 prevention***  *--* Changing emergency-medical service procedures (ambulances, paramedics)  -- Re-locating hospital-based ambulatory clinics, cancer treatments, etc.  -- Limiting access to health facilities  -- Changing hospital-discharge procedures  -- Changing long-term care procedures  -- Changing home and community care procedures  ***- Service planning for COVID-19 treatment***  *--* Scaling up/down testing capacity  -- Scaling up/down emergency-room capacity  -- Scaling up/down ICU capacity  -- Scaling up/down post-ICU recovery capacity (e.g., hospital beds)  -- Scaling up/down palliative-care capacity  -- Scaling up/down COVID-19 sequelae management capacity  -- Scaling up/down capacity to manage the pandemic-related impacts on health more generally (e.g., mental  health and addictions)  -- Surge-management models  -- Triage protocols  -- Infection prevention and control measures in health facilities  -- Death certification  -- Handling dead bodies  ***- Service planning for the ongoing management of other conditions***  -- Changing acute care surgery and trauma-care procedures  -- Changing cancer-treatment procedures  -- Changing reproductive care  -- Delaying return visits, elective procedures, etc. ***- Infrastructure planning and resource allocation***  -- Personal protective equipment (under shortage conditions), including N95 respirators for health workers  -- Ventilators for sick COVID-19 patients  -- Medications and other technologies (under shortage conditions due to disrupted supply chains)  -- Remote monitoring  -- Virtual visits  ***- Workforce planning (including workforce shortages management) and development***  -- Recruitment  -- Role extensions  -- Training in new procedures  -- Replacements when sick  -- Re-deployment  -- Supports to unpaid caregivers  -- Volunteer engagement  -- Self-management support  ***-*** ***Service planning for ‘return to normal’***  -- Sequencing of services re-starting, by sector, conditions, treatments (including diagnostics), and populations  -- Wait-lists management |
| **Public health measures: Broader public health measures** | ***- Risk stratification***  -- Stratifying the population by risk of infection ***- Outbreak management***  -- Locations (essential services or others)  -- Rapid-response mechanisms ***- Pandemic tracking***  -- Levels of re-emergence that trigger action |
| **Public health measures: Infection control** | ***- Screening***  -- Targets  -- Methods  -- Locations (and *frequency if applicable)* ***- Quarantining of exposed or potentially exposed individuals***  -- Voluntary or imposed ***- Testing***  -- Optimizing testing across different types of individuals, settings and timing options  -- Methods used in the test  -- Methods (type of specimen*)*  -- *Methods (site from where specimen is taken)*  -- *Speeding results* ***- Isolation of suspected or confirmed cases - Contact tracing - Susceptibility tracking - Antibody testing - Antibody test usage*** |
| **Public health measures: Infection prevention** | ***- Personal protection***  -- Washing hands  -- Wearing masks  -- Wearing personal protective equipment  -- Disinfecting surfaces and facilities  -- Physical distancing  -- Temporal distancing  -- Altering sexual activities  -- Public-focused behaviour-change supports for the above  -- Health worker and essential worker-focused behaviour change supports for the above  -- Other  ***- Service limitations***  ***- Vaccination***  -- Safety and efficacy of two doses of the same vaccine if two doses are recommended (or a single dose if a  single dose is recommended)  -- Safety and efficacy of one dose of one vaccine and a second dose of a different vaccine (i.e., mix and match or  heterologous prime and boost)  -- Safety and efficacy of one dose if two doses are recommended  -- Safety and efficacy of three doses if two doses were recommended before the emergence of variants  -- Real world effectiveness  -- Efficacy/effectiveness by population segment  -- Vaccine roll-out (see health-system arrangements)  -- Supporting discovery of a vaccine to prevent COVID-19 in general, and for specific population groups |

Robert Koch Institute (RKI) Control COVID

| **Category** | **Sub-category** |
| --- | --- |
| **Basic measures** | ***- "AHA+L" (distance - hand hygiene - mask + ventilation) - Vaccination - General wearing of masks in health care settings and nursing homes - Case identification and case isolation - Contact tracing and quarantine - Identification of case chains and clusters - Reduce mobility and contacts - Corona alert app - National testing strategy*** |
| **Measures by setting** | ***- Closure - Protection or hygiene concept - Cohorting - Prohibition and cancellation - Reduction of TN number/visitor number/user number/etc. - Mandatory testing - Distance learning*** |
| **Settings** | ***- Indoor gatherings - Retirement and nursing homes - Bars / Clubs - Businesses/companies  - Gastronomy - Universities & colleges - Secondary and vocational schools  - Public transport - Child care institutions & elementary schools - "Religious communities/Religious gatherings" - Theatre, cinema, museums - Hairdresser, cosmetics, personal care - Retail - Outdoor gatherings - Long distance passenger transport - Hotels - Parks and playgrounds*** |

Complexity Science Hub COVID-19 Control Strategies List (CCCSL)

| **Category** | **Sub-category** |
| --- | --- |
| **Case identification, contact tracing and related measures** | ***- Activate case notification - Airport health check - Border health check - Enhance detection system - Isolation of cases - Quarantine - Restricted testing - Surveillance - Tracing and tracking*** |
| **Environmental measures** | ***- Enhance hygiene conditions - Environmental cleaning and disinfection*** |
| **Health and public health capacity** | ***- Adapt procedures for patient management - Develop new health services - Enhance laboratory testing capacity - Increase availability of PPE - Increase healthcare workforce - Increase in medical supplies and equipment - Increase isolation and quarantine facilities - Increase patient capacity - Personal protective measures - Repurpose hospitals - Research - Secure future access to anti-Covid19 medication and vaccine*** |
| **Resource allocation** | ***- Activate or establish emergency response - Crisis management plans - Economic measure to stimulate consumption - Measures to ensure security of supply - Police and army interventions - Provide international help - Receive international help - The government provide assistance to vulnerable populations*** |
| **Risk communication** | ***- Actively communicate with healthcare professionals - Actively communicate with managers - Educate and actively communicate with the public - Travel alert and warning*** |
| **Social distancing** | ***- Closure of educational institutions - Indoor and outdoor gathering restriction - Indoor gathering restriction - Measures for public transport - Measures for special populations - Outdoor gathering restriction - Special measures for certain establishments - Work safety protocols*** |
| **Travel restriction** | ***- Airport restriction - Border restriction - Cordon sanitaire (Establishment of a geographic containment zone, typically monitored by authorities, to restrict***  ***movement into and out of an infected area.) - Individual movement restrictions - National lockdown*** |
| **Returning to normal life** | ***- Access to non-essential/critical healthcare services - Actively communicate with managers - Educate and actively communicate with the public - Exemption of quarantine - Lift airport restrictions - Lift border restrictions - Lift measures to protect vulnerable populations - Lift personal protective measures - Lift restriction on individual movements - Lift restriction on indoor and outdoor gatherings - Lift restriction on indoor gatherings - Lift restriction on outdoor gatherings - Lift restriction on public transports - Lift travel restriction - Phase out emergency management - Re-opening of educational institutions - Resume export of medical and personal protective equipment*** |

Oxford COVID-19 Government Response Tracker

| **Category** | **Sub-category** |
| --- | --- |
| **Containment and closure policies** | ***- C1_School closing  - C2_Workplace closing - C3_Cancel public events - C4_Restrictions on gatherings - C5_Close public transport - C6_Stay at home requirements - C7_Restrictions on internal movement - C8_International travel controls*** |
| **Health system polices** | ***- H1_Public information campaigns - H2_Testing policy - H3_Contact tracing - H4_Emergency investment in healthcare - H5_Investment in vaccines - H6_Facial Coverings - H7_Vaccination Policy - H8_Protection of elderly people*** |
| **Vaccination policies** | ***- V1_Vaccine prioritisation (summary)***  ***- V2_Vaccine eligibility/availability - V3_Vaccine financial support*** |

CoronaNet COVID-19 Government Response Event Dataset

| **Category** | **Sub-category** |
| --- | --- |
| **Border restriction** | ***- Travel_mechanism***  -- int_restrict_flights. Takes a value of 1 if flights are restricted.  -- int_restrict_border. Takes a value of 1 if travel through land borders is restricted.  -- int_restrict_all. Takes a value of 1 if all kinds of transport across borders are restricted.  -- int_restrict_NA. Takes a value of 1 if the type of border restriction is not specified  -- int_restrict_cruises. Takes a value of 1 if cruise ships are restricted.  -- int_restrict_ferries. Takes a value of 1 if ferries are restricted.  -- int_restrict_ports. Takes a value of 1 if travel through seaports is restricted.  -- int_restrict_trains. Takes a value of 1 if trains are restricted.  -- int_restrict_buses. Takes a value of 1 if buses are restricted. |
| **Business restrictions** | ***- Type of business*** (very long list; not copied) ***- Type of measure***  -- biz_hygiene. Takes a value of 1 if hygiene and sanitation measures are required as a condition of business operating.  -- biz_hours. Takes a value of 1 if the number of working hours is limited as a condition of business operating.  -- biz_work_home. Takes a value of 1 if the number of employees and working hours are limited as a condition of  business operating.  -- biz_meeting. Takes a value of 1 if the size of business meetings is limited as a condition of business operating.  -- biz_social_distance. Takes a value of 1 if keeping a distance of at least 6ft or 1.5 meters apart is required as a  condition of business operating.  -- biz_mask. Takes a value of 1 if mask wearing is required as a condition of business operating.  -- biz_temperature. Takes a value of 1 if temperature checks are required as a condition of business operating.  -- biz_health_cert. Takes a value of 1 if health certificates are required as a condition of business operating.  -- biz_health_q. Takes a value of 1 if health questionnaires are required as a condition of business operating.  -- biz_num_cust. Takes a value of 1 if the number of customers is limited as a condition of business operating.  -- biz_store_size. Takes a value of 1 if the size of stores is limited as a condition of business operating.  -- biz_cont_trace. Takes a value of 1 if contact tracing is required as a condition of business operating.  -- biz_cond_other. Takes a value of 1 if other conditions are required as a condition of business operating. |
| **Health resources** | ***- Type_health resources*** |
| **Health monitoring** | ***- Type_health_mon*** |
| **Health testing** | ***- Type_health_testing*** |
| **Mask** | ***- Type of measure***  -- mask_public. Takes a value of 1 if there is a policy for wearing masks inside public buildings.  -- mask_everywhere. Takes a value of 1 if there is a policy for wearing masks in all public spaces.  -- mask_business. Takes a value of 1 if there is a policy for wearing masks inside private businesses (e.g. supermarkets).  -- mask_primary _school. Takes a value of 1 if there is a policy for wearing masks inside primary schools (generally for  children ages 10 and below).  -- mask_sec_school. Takes a value of 1 if there is a policy for wearing masks inside secondary schools (generally for  children ages 10 and to 18).  -- mask_transport. Takes a value of 1 if there is a policy for wearing masks inside public transportation.  -- mask_unspec. Takes a value of 1 if there is an unspecified mask wearing policy.  -- mask_preschool. Takes a value of 1 if there is a policy for wearing masks inside preschools or childcare  facilities(generally for children ages 5 and below).  -- mask_higher _ed. Takes a value of 1 if there is a policy for wearing masks inside higher education institutions (i.e.  degree granting institutions) |
| **Mass gatherings** | ***- Type of measure***  -- curfew_length. Takes a value of 1-24 (hours).  -- number_mass. Takes a value of 1 if there is the most stringent mass gathering restriction and 0 if there is no mass  gathering restriction.  -- cancel_annual_event. Takes a value of 1 if there is a cancellation of a recreational or commercial event.  -- prison_pop. Takes a value of 1 if there is a policy regarding reduction of prison population.  -- postpone_ann_event. Takes a value of 1 if there is a postponement of a recreational or commercial event.  -- postpone_rec_event. Takes a value of 1 if there is a postponement of an annually recurring event.  -- private_event. Takes a value of 1 if events at private residencies are restricted.  -- event_no_audience. Takes a value of 1 if events are allowed to occur without an audience. |
| **School restriction** | ***- Type of school***  -- preschool  -- primary_school  -- secondary_school  -- higher_ed ***- Type of measure***  -- school_clean. Takes a value of 1 if regular cleaning and sanitary procedures are required as a condition of schools  operating.  -- school_other. Takes a value of 1 if other conditions are required as a condition of schools operating.  -- school_num. Takes a value of 1 if the number of people on the school premises is limited as a condition of schools  operating.  -- school_type_pers. Takes a value of 1 if the types of people on the school premises are limited as a condition of  schools operating.  -- school_health_q. Takes a value of 1 if health questionnaires are required as a condition of schools operating.  -- school_special_student. Takes a value of 1 if special provisions exist for all students in a school (e.g. students in  primary school do not have to social distance).  -- school_special_teacher. Takes a value of 1 if special provisions exist for how teaching is done (e.g. teachers must  tele-teach).  -- school_temp. Takes a value of 1 if temperature checks are required as a condition of schools operating.  -- school_health_monitoring. Takes a value of 1 if other health monitoring measures are required as a condition of  schools operating. |
| **Social distancing** | ***- Type of measure***  -- social_distance. Takes a value of 1 if there is a policy for keeping a distance of at least 6 feet or 1.5 meters apart.  -- distance_other. Takes a value of 1 if there is a policy for keeping a distance of some other distance not listed above.  -- buses. Takes a value of 1 if there are restrictions on ridership of buses. other_transport. Takes a value of 1 if there are restrictions on ridership of other forms of public transportation.  -- private_transport. Takes a value of 1 if there are restrictions on ridership private vehicles in public circulation.  -- subways. Takes a value of 1 if there are restrictions on ridership of subways and trams.  -- trains. Takes a value of 1 if there are restrictions on ridership of trains. |

Health Intervention Tracking for COVID-19 (HIT-COVID)

| **Category** | **Sub-category** |
| --- | --- |
| **Restrictions of travel and movement** | ***- Border closures - Limiting movement within administrative unit borders - Household confinement*** |
| **Social and physical distancing measures** | ***- Closures of public institutions and public areas - Closures of non-public institutions and areas - Limiting gatherings*** |
| **Surveillance and response measures** | ***- Symptom screening at borders - Testing individuals - Contact tracing - Quarantine and home-isolation*** |
| **Other measures** | ***- Military and police deployment - State of emergency - Mandated face mask use*** |

European Center for Disease Control (ECDC) Taxonomy

| **Category** | **Sub-category** |
| --- | --- |
| **Individual level** | ***- Physical distancing - Respiratory hygiene - Hand hygiene - Face masks - Face shields and goggles - Gloves*** |
| **Population level** | ***- Limiting close physical inter-personal interactions***  -- Isolation of symptomatic cases not requiring hospitalisation  -- Quarantining of contacts  -- Shielding medically- and sociably-vulnerable populations  -- Recommending ‘social bubbles’  -- Long-term care facilities (LTCFs)  -- Prisons  -- Migrants and refugees residing in reception and detention centres  -- Limiting size of gatherings  -- Measures at the workplace, including teleworking  -- Closure of non-essential businesses  -- School closures  -- Stay-at-home measures |
| **Environmental level** | ***- Environmental cleaning - Ventilation*** |
| **Travel-related measures** | ***- International travel restrictions and border closures - Measures on conveyances and travel hubs - Travel advice - Screening at points of entry at national borders - Quarantine of passengers - Domestic travel restrictions*** |

ACAPS COVID-19 Government Measures Dataset

| **Category** | **Sub-category** |
| --- | --- |
| **Movement restrictions** | ***- Additional health or other document requirements upon arrival - Border checks  - Border closure - Complete border closure - Checkpoints within the country - International Flights suspension - Domestic travel restrictions  - Visa restrictions - Curfews - Surveillance and monitoring*** |

COVID-19 Hierarchy of Control

| **Category** |
| --- |
| **Administrative controls** |
| **Community protective equipment** |
| **Elimination** |
| **Engineering controls** |
| **Personal protective equipment** |

Polisena et al. (2021)

| **Category** | **Sub-category** |
| --- | --- |
| **Public health communication and education** | ***- Hand hygiene***  ***- Respiratory etiquette***  ***- Environmental cleaning (home/personal environment)***  ***- Voluntary self-isolation***  ***- Voluntary home quarantine***  ***- Use of masks*** |
| **Public health orders** | ***- Environmental cleaning (public spaces)***  ***- Physical distancing measures***  ***- School closures***  ***- University closures***  ***- Childcare closures***  ***- Cancellation of mass gatherings***  ***- Assisted living facilities***  ***- Workplace (remote work)***  ***- Healthcare settings***  ***- Community/faith-based organisations***  ***- Retail/commerce restrictions***  ***- Remote and isolated communities***  ***- Border and travel measures***  ***- Case and contact or tracing management measures***  ***- Justice services***  ***- Government services***  ***- Social services*** |

[Wang and Mao (2021)](https://www.ncbi.nlm.nih.gov/pmc/articles/PMC8178944/)

| **Category** | **Sub-category** |
| --- | --- |
| **Movement restriction** | ***- Banning entry of all foreign nationals who had been to China's Hubei province in the past two weeks - Banning entry of foreign nationals from other high-risk areas besides Hubei, China - Banning entry of all foreign nationals - Mandatory 14-day quarantine on all passengers arriving from Hubei, China - Mandatory 14-day quarantine on all passengers arriving from all parts of the world - Domestic travel restrictions between cities or provinces*** |
| **Physical and social distancing measures** | ***- Confirmed cases and close contacts***  -- Mandatory isolation of confirmed cases and their close contacts  -- Tracking COVID-patients and their close contacts  -- Develop mobile applications to track COVID-19 patients and close contacts  -- Patients with different degrees of symptoms treated in different locations ***- Suspected cases***  -- Self-isolation of those from high-risk areas  -- Self-isolation for symptomatic people or for those living with symptomatic people  -- Temperature check in public spaces  -- Virus testing **- The general public**  -- National stay at home orders  -- School closure or delayed start of school semesters  -- Working from home  -- Non-essential shops and services closed  -- Curfew  -- Cancellation of public events |
| **Personal measures** | ***- Hand washing - Ventilation - Social distancing - Wear a face mask in public for healthy people*** |
| **Special protection measures for special populations and vulnerable groups** |  |
| **Public health resources strategies** | ***- Supply medical goods and materials - Provide healthcare staff protective gears - Increase hospital beds for treating COVID-19 patients - Dispatch medical teams from other parts of the country to epidemic areas - Military support*** |

Travel measures review (Burns et al. 2021)

| **Category** | **Sub-category** |
| --- | --- |
| **Travel restrictions stopping or reducing cross-border travel** | ***- Closure of national borders to entry and/or exit - Isolation of travellers (entry/exit) - International travel restrictions/bans***   -- Denial of entry and/or exit on the basis of nationality, travel history, health status or other characteristics  -- Partial suspension of cross-border travel via land and/or air and/or sea |
| **Entry/exit screening at national borders** | ***- Symptom/exposure-based screening (e.g. temperature measurement, thermography, health questionnaire/declaration cards, passive observation, physical examination) - Vaccination/immunity status-based screening - Test-based screening (e.g. via rapid antigen or Reverse Transcription Polymerase Chain Reaction (RT-PCR) tests) - Other screening approaches (e.g. use of sniffer dogs)*** |
| **Quarantine or isolation of travellers crossing national borders** |  |
| **Multi-component travel-related control measures, including any combination of the above measures** |  |

School measures review (Krishnaratne et al. 2021)

| **Category** | **Sub-category** |
| --- | --- |
| **Measures reducing the opportunity for contacts** | ***- Phased reopening of schools - Reduced cohort size - Staggered start/end time - Alternating attendance - Only allowing schooling in-person for certain grades/students*** |
| **Measures making contacts safer** | ***- Face masks - Handwashing interventions - Cleaning interventions - Modifying activities in the school setting - Ventilation interventions - Combined measures to make contacts safer*** |
| **Surveillance and response measures** | ***- Mass testing and isolation measures - Symptom-based screening and quarantine measures*** |
| **Multi-component measures** | ***- A combination of multiple measures including: reduced cohort size, face masks, handwashing interventions, modifying activities in the school setting, cleaning, testing, and quarantine*** |

**Figure S1** Development process towards initial conceptual framework of PHSM

**­­­­**

**
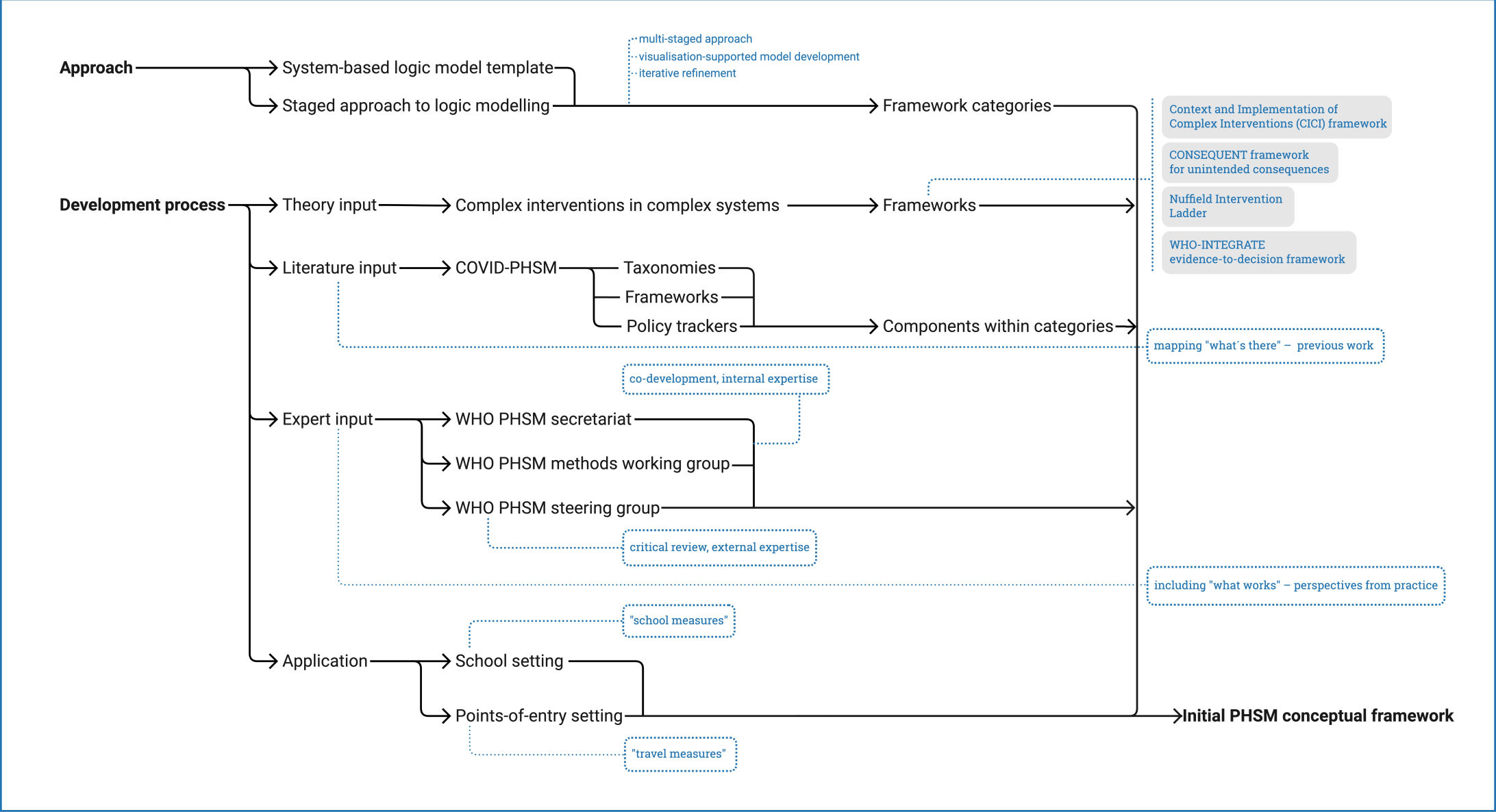
**

**Figure S2** An initial conceptual framework of public health and social measures during health emergencies: framework categories and their components (population, setting, contextual factors and context-specific, equity-sensitive decision-making expanded)

**
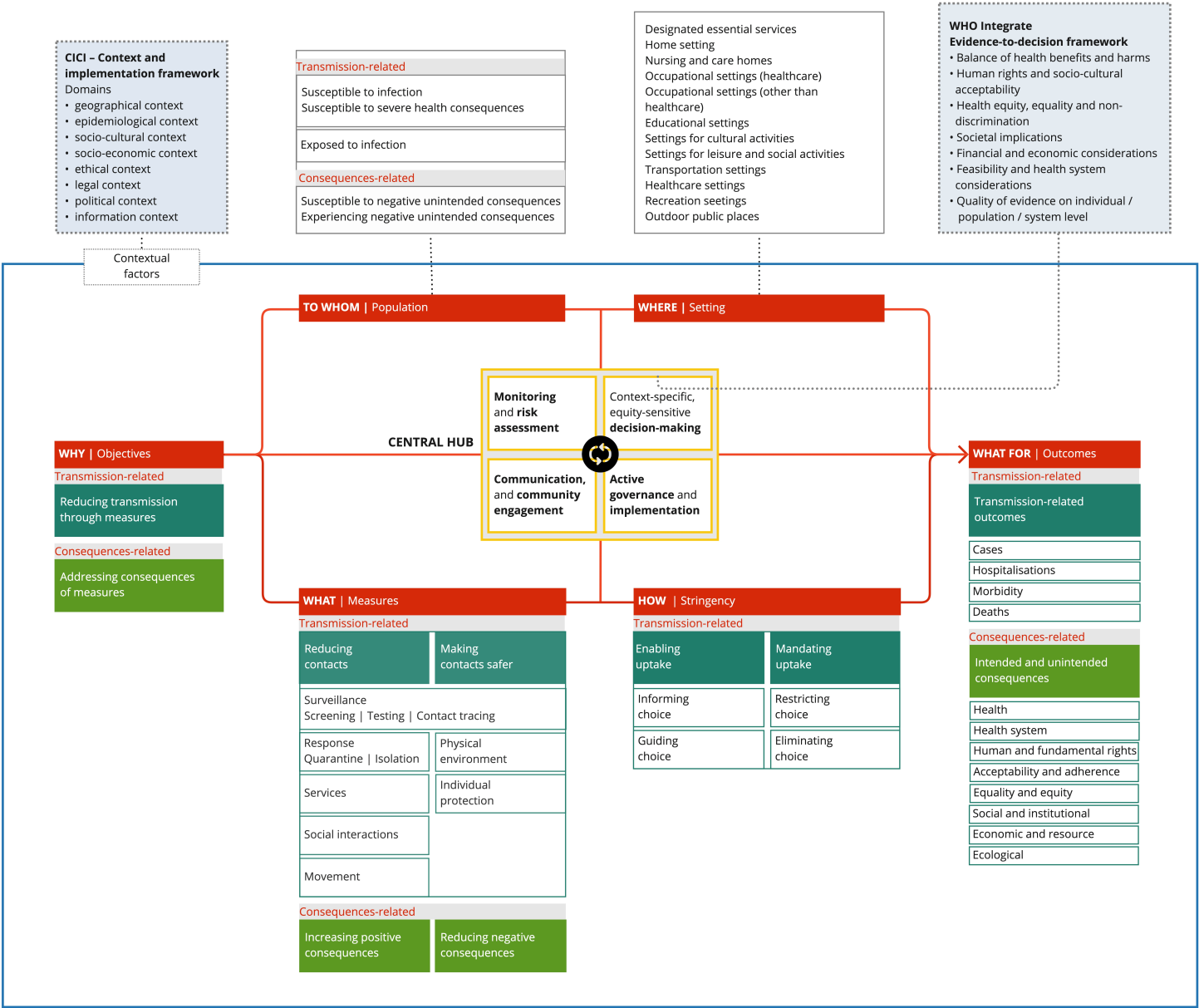
**

**Table S1** Classification of measures for schools during COVID-19

The aim of measures for schools is to maintain schools open to the largest degree possible. The classification of modes of enactment ranging from informing choice to eliminating choice of individuals within the school setting refers to the *maintenance of essential school services* as well as *other school activities in-person* for a *majority of students*. The focus of these measures is on students, but other populations - importantly headmasters, teachers and other school staff but also parents - play a role, too. Essential school services encompass education across the full range of subjects, school health services and school meals, as well as travel to and from school. The mode of enactment may target *individuals* (i.e. students, teachers, school staff, parents) or *institutions* (i.e. decision-makers in these institutions); for most measure categories, both levels are being targeted. Notably, with physical environment measures usually operating at a group level (i.e. classrooms, cafeterias, whole schools), the mode of enactment primarily targets the institution.

| **Measure** | **Mode of enactment** | **Examples** |
| --- | --- | --- |
| Surveillance | Informing choice | - Providing information on symptoms of COVID-19 - Providing information on where to get tested - Providing information on decision routes when noticing symptoms (e.g. when and where to get a PCR test) - Recommending voluntary testing after school holidays - ... |
|  | Guiding choice | - Providing tests to students and school staff free of charge - Providing access to official testing centres - ... |
|  | Restricting choice | - Requiring a form that confirms screening for symptoms and/or a negative test result obtained from testing at home before entering a school - Allowing student access to school premises only with proof of a negative test and allowing for home schooling as an alternative - Restricting access to school premises to tested **or** vaccinated school staff - ... |
|  | Eliminating choice | - Requiring regular testing at schools (e.g. three times a week using rapid antigen tests or twice a week using PCR pool testing) and not allowing for home schooling as an alternative - Excluding students from participating in certain essential activities based on fulfilment of testing requirements (e.g. music class) - Allowing access to school premises to school staff only with proof of a negative test - ... |
| Response | Informing choice | - Providing information on quarantine and isolation (e.g. duration, possibility to get tested) - Recommending voluntary quarantine after contact with a case - Recommending “stay at home if you are sick” with COVID-like symptoms - … |
|  | Guiding choice | - Introducing school-based penalties (e.g. disciplinary measures) for not adhering to stay-at-home policies - Offering alternative childcare for sick children - Additional sick leave for parents looking after sick or quarantined children - … |
|  | Restricting choice | - Requiring all close contacts of a case in the school setting to test daily - … |
|  | Eliminating choice | - Requiring students with COVID-like symptoms to stay at home for two days and only allowing access after a negative test result - Quarantining close contacts of a case in the school setting (individual contact, entire class) - Isolating infected students or staff - ... |
| Services | Informing choice | - Providing information on how to safely use services offered in educational settings - Issuing recommendations (e.g. on billboards) on how to safely use services offered in educational settings - ... |
|  | Guiding choice | - Introducing staggered school lunch servings - Modifying school nurse services (e.g. appointment only and longer service hours to prevent overcrowded waiting rooms) - … |
|  | Restricting choice | - Modifying school activities (e.g. cancellation of physical education classes; modification of music classes) - Restricting the number of people in certain areas (e.g. school cafeterias) - Alternating attendance (alternating weeks, alternating days) in classrooms - Cohorting students (e.g. through staggered start/end dates) - ... |
|  | Eliminating choice | - Closing school cafeterias and shops - Extending holidays - Instituting fully digital learning for selected cohorts of students (e.g. older students) - Closing schools - ... |
| Social interactions | Informing choice | - Providing information on how to reduce social interactions as a protective strategy - Encouraging students, teachers and school staff to consider social distancing (e.g. lunch with small and fixed group of people, afternoon activities with a limited circle of school friends) - ... |
|  | Guiding choice | - Setting up “learning groups” in a way that respects friendships and animosities within a class - … |
|  | Restricting choice | - Restricting school-related social events to students only or cohorts of students only (e.g. Christmas party, school-leaving party) - Restricting the number of people having access to school-related social events - Restricting access to campus to students and school staff only - ... |
|  | Eliminating choice | - Banning school/cohort-level social events (e.g. Christmas party, school-leaving party) - Banning gatherings of student on school grounds - ... |
| Movement | Informing choice | - Providing information on how to safely move to and from school and on school premises - Recommending walking or cycling to school - ... |
|  | Guiding choice | - Incentivising students and school staff to walk/bike to school (e.g. class competitions) - Hiring additional school buses - ... |
|  | Restricting choice | - Restricting the number of people in certain locations (e.g. hallways, school buses) - Setting up cohort-dependent entry to the school building and/or one-way walking systems - ... |
|  | Eliminating choice | - Stopping public transport to school (e.g. school buses) - … |
| Physical environment | Informing choice | - Providing information about cleaning, disinfection and ventilation - Recommending regular cleaning and ventilation - ... |
|  | Guiding choice | - Providing materials for routine disinfection on every desk - Providing and supporting the maintenance of air purifiers - Setting up a seating area for outdoor lunch - Making available additional facilities to enable safe physical education classes - Providing and/or upgrading water and sanitation facilities - Introducing infrastructural measures to facilitate physical distancing between cohorts (e.g. ropes, separate entrances, floor labelling) - ... |
|  | Restricting choice | - Allowing teaching only in rooms with sufficient and appropriate ventilation (e.g. resulting in modified timetables) - Allowing use of rooms for afternoon activities only after cleaning - … |
|  | Eliminating choice | - Closing schools due to insufficient and inadequate infrastructure - ... |
| Individual protection | Informing choice | - Providing information on physical distancing, cough and sneeze etiquette, proper hand hygiene and mask wearing - Recommending to wear masks (community masks, surgical masks, FFP2 masks) in crowded areas - ... |
|  | Guiding choice | - Providing adequate masks at school for free - Introducing a school policy on mask wearing with school-based penalties for non-adherence (e.g. letter to parents) - Setting up a design competition or photo contest related to individual protection measures - ... |
|  | Restricting choice | - Restricting access to certain areas to individuals wearing masks - Stopping activities that cannot be done without appropriate individual protection (e.g. playing the flute) - ... |
|  | Eliminating choice | - Mandating surgical masks for students and school staff - Mandating FFP2 masks for visitors at school - ... |

**Table S2** Classification of measures for international travel and points of entry during COVID-19

The aim of international travel measures is to reduce the risk of transmission through or during travel between countries via air, land or sea, thereby avoiding or delaying importations/exportations of cases. The classification of modes of enactment ranging from informing choice to eliminating choice of individuals in this setting refers to the ability of individuals or groups to freely travel between countries, as well as to freely use a range of services (e.g. meals) and opportunities (e.g. having accompanying relatives at the departure/arrival areas) during travel and/or at the point of entry. The mode of enactment may target *individuals* (i.e. travellers, stewardesses, train station staff) or *institutions* (i.e. decision-makers at points of entry or on planes, ships, buses and trains crossing borders); for most measure categories, both levels are being targeted. Notably, with physical environment measures usually operating at a group level (i.e. restaurants, wash rooms, waiting areas), the mode of enactment primarily targets the institution.

| **Measure** | **Mode of enactment** | **Examples** |
| --- | --- | --- |
| Surveillance | Informing choice | - Providing information on symptoms of COVID-19 and benefits of screening before traveling - Providing information on when (e.g. how many hours before travel) and where to get tested before/after entering a country - Recommending a voluntary test at points of entry - … |
|  | Guiding choice | - Offering free-of-charge voluntary PCR-testing at points of entry - Offering (extra) free-of-charge meals/snacks during travel for those showing a negative test result - … |
|  | Restricting choice | - Requiring all international travellers to be symptom-free or to have a negative PCR-test result before boarding for travel - Requiring all international travellers to have a negative PCR-test or a proof of immunity (full vaccination or recovery) to avoid quarantine upon entering a country - … |
|  | Eliminating choice | - Banning access to certain travel services (e.g. restaurant at airport or train station) without a negative PCR-test - Requiring all international travellers to have proof of immunity and a negative PCR-test upon entering a country - … |
| Response | Informing choice | - Providing information on quarantine and isolation when entering a country (e.g. duration and conditions of early termination) - Recommending to voluntarily quarantine when entering a country - … |
|  | Guiding choice | - Offering free-of-charge hotel rooms for voluntary quarantine at points of entry - Offering free-of-charge services to those in a voluntary quarantine (e.g. testing visits, food supplies) - … |
|  | Restricting choice | - Requiring all international travellers to quarantine upon entering a country unless presenting with a negative PCR-test or being fully vaccinated - … |
|  | Eliminating choice | - Requiring all international travellers to quarantine upon entering a country despite presenting with a negative PCR-test result or a proof of immunity (full vaccination or recovery) - … |
| Services | Informing choice | - Providing information on how to safely use services offered during international travel or at points of entry - Making recommendations on the safe use of services during international travel or at points of entry (e.g. take-away rather than stay-in food) |
|  | Guiding choice | - Offering discounts when ordering take-away food at restaurants at points of entry - Offering visa fee discounts when travel is postponed to a later date - … |
|  | Restricting choice | - Restricting the number of individuals in certain areas at points of entry (e.g. restaurants and shops at airports) - Reducing the number and types of services operating at airports or train stations (e.g. shops, restaurants) - Reducing the availability of visa appointments at visa centres/embassies for entering a country - … |
|  | Eliminating choice | - Closing all restaurants and shops at points of entry - Stopping to serve meals during short international travels - Closing visa centres - … |
| Social interactions | Informing choice | - Providing information on how to reduce social interactions at points of entry - Recommending social distancing at points of entry (e.g. avoid large groups in restaurants) - … |
|  | Guiding choice | - Sending an alert for social distancing on a dedicated app when a close contact is detected at points of entry - … |
|  | Restricting choice | - Restricting access to airport, port, bus or train station buildings to allow for only 1 person accompanying the traveller (e.g. relatives in departure and arrival areas) - Restricting the number of individuals in washing rooms - … |
|  | Eliminating choice | - Prohibiting access to airport, port, bus or train station buildings to those not traveling (e.g. relatives in departure and arrival areas) - Blocking washing rooms for use by one person at a time - … |
| Movement | Informing choice | - Providing information on how to safely travel to and from a country - Recommending to reduce international travel - … |
|  | Guiding choice | - Allowing for free rebooking to postpone travel during a high-community transmission period to a low-community transmission period - …. |
|  | Restricting choice | - Reducing international travel options (e.g. limiting international arrivals to a small number of well-controlled airports) - Reducing the number of international flights (generic, country-specific) - … |
|  | Eliminating choice | - Banning/suspending travel to/from countries with circulating variants of concern - … |
| Physical environment | Informing choice | - Providing information about cleaning, disinfection and ventilation - Recommending regular cleaning and ventilation - … |
|  | Guiding choice | - Offering free-of-charge seats with comfortable cushions in waiting areas at points of entry (e.g. airports) with adequate ventilation - Providing sanitation materials/infrastructure at all airport gates - Introducing infrastructural measures to facilitate physical distancing at points of entry (e.g. ropes, floor labelling) - … |
|  | Restricting choice | - Stopping the use of certain areas at points of entry (e.g. restaurants) unless adequately ventilated and disinfected - Removing or blocking directly adjacent seats in waiting areas at points of entry - … |
|  | Eliminating choice | - Closing services at points of entry (e.g. restaurants, shops) due to lack of adequate infrastructure - … |
| Individual protection | Informing choice | - Providing information on physical distancing, cough and sneeze etiquette, proper hand hygiene and mask wearing at points of entry - Recommending to use masks (e.g. surgical, FFP2 masks) at points of entry - … |
|  | Guiding choice | - Offering free surgical or FFP2 masks to travellers at points of entry for voluntary use - Providing free hand sanitizers to travellers for voluntary use before boarding the aircraft - … |
|  | Restricting choice | - Restricting access to certain areas at points of entry to travellers wearing masks (e.g. restaurants) unless fully vaccinated or recovered or presenting a negative PCR test - … |
|  | Eliminating choice | - Requiring all travellers to wear FFP2 masks at points of entry - Requiring all travellers to sit one seat apart in waiting areas at points of entry - … |
